# Phenethyl Ester of Gallic Acid Ameliorates Experimental Autoimmune Encephalomyelitis

**DOI:** 10.3390/molecules27248770

**Published:** 2022-12-10

**Authors:** Goran Stegnjaić, Antonios D. Tsiailanis, Milica Lazarević, Vasileios K. Gkalpinos, Neda Djedovic, Thomas Antoniou, Suzana Stanisavljević, Mirjana Dimitrijević, Miljana Momčilović, Đorđe Miljković, Andreas G. Tzakos, Bojan Jevtić

**Affiliations:** 1 Department of Immunology, IBISS, University of Belgrade, 11060 Belgrade, Serbia; 2 Section of Organic Chemistry & Biochemistry, Department of Chemistry, University of Ioannina, 54110 Ioannina, Greece; 3 Institute of Materials Science and Computing, University Research Center of Ioannina (URCI), 45110 Ioannina, Greece

**Keywords:** experimental autoimmune encephalomyelitis, T cells, macrophages, microglia, cytokines

## Abstract

Gallic acid is a phenolic acid present in various plants, nuts, and fruits. It is well known for its anti-oxidative and anti-inflammatory properties. The phenethyl ester of gallic acid (PEGA) was synthesized with the aim of increasing the bioavailability of gallic acid, and thus its pharmacological potential. Here, the effects of PEGA on encephalitogenic cells were examined, and PEGA was found to modulate the inflammatory activities of T cells and macrophages/microglia. Specifically, PEGA reduced the release of interleukin (IL)-17 and interferon (IFN)-γ from T cells, as well as NO, and IL-6 from macrophages/microglia. Importantly, PEGA ameliorated experimental autoimmune encephalomyelitis, an animal model of chronic inflammatory disease of the central nervous system (CNS)—multiple sclerosis. Thus, PEGA is a potent anti-inflammatory compound with a perspective to be further explored in the context of CNS autoimmunity and other chronic inflammatory disorders.

## 1. Introduction

Autoimmune reactivity against the central nervous system (CNS) plays a dominant role in the pathogenesis of multiple sclerosis—a chronic inflammatory, demyelinating, and neurodegenerative disease of the CNS [1]. Accordingly, the majority of the drugs available for the treatment of patients with multiple sclerosis are potent immunomodulators or immunosuppressors that limit immune responses directed against the CNS. These drugs are effective in reducing the progression of the disease and lessening its symptoms, but they are not devoid of side effects, and none of them cure multiple sclerosis [2]. Thus, there is room for the improvement of multiple sclerosis therapy, and natural products are conceivable candidates to be exploited as an alternative strategy for effective treatment. [3]. Indeed, numerous studies on preclinical models of multiple sclerosis provided promising results on the possibility of using natural products to treat CNS autoimmunity [4].

Gallic acid (GA), a plant secondary metabolite present in various plants, nuts, and fruits, has been attributed to strong anti-inflammatory properties [5]. The anti-inflammatory effects of GA have been associated with its ability to interfere with MAPK and NF-κB signaling, thus limiting the activation and effector properties of immune cells [5]. GA has been shown to inhibit interleukin-(IL)-17–IL-17-receptor interactions in psoriasis-like disease in mice [6], as well as to reduce IL-17 production in nasal and colon tissue in animal models of rhinitis and ulcerative colitis, respectively [7,8]. It also reduced mRNA levels of interferon (IFN)-γ and IL-17 in lymph nodes in the murine model of atopic dermatitis [9]. Additionally, GA was shown to inhibit dendritic cells maturation in vitro [10]. IL-17-producing T helper (Th)17cells are among the most important pathogenic populations in multiple sclerosis, while dendritic cells are crucial for the activation of naïve autoreactive CD4^+^ T cells, including Th17 [1]. Thus, the reported effects of GA on IL-17 production and dendritic cell maturation are highly relevant for pharmacological intervention in CNS autoimmunity. Indeed, GA has been shown toameliorates experimental autoimmune encephalomyelitis (EAE) [11]. However, the polar carboxylic acid group reduces the capacity of GA to permeate through biological membranes, thus limiting its bioavailability and lessening its pharmaceutical potency. To overcome these limitations, we applied a methodology on the frame of which we developed lipophilic derivatives of phenolic acids as we have previously reported [12,13]. Through this derivatization, the resulting compounds showed enhanced biological activity including anti-oxidative potency and prevention of H_2_O_2_-induced DNA damage and apoptosis [12,13]. Along these lines, phenethyl ester derivatives showed the best results in improving the cell permeability of the compounds in question [13]; herein, we used phenethyl ester of gallic acid (PEGA).

Having in mind the above presented facts, we have evaluated PEGA in the setting of CNS autoimmunity both in vitro and in vivo. The effects of PEGA on the release of IFN-γ, IL-17 from T cells, IL-6, tumor necrosis factor (TNF), and NO by macrophages/microglia were examined in vitro. The effectiveness of PEGA in vivo was investigated in EAE.

## 2. Results

### 2.1. PEGA Affects Popliteal Lymph Node Cells (PLNC)

**Figure 1:**
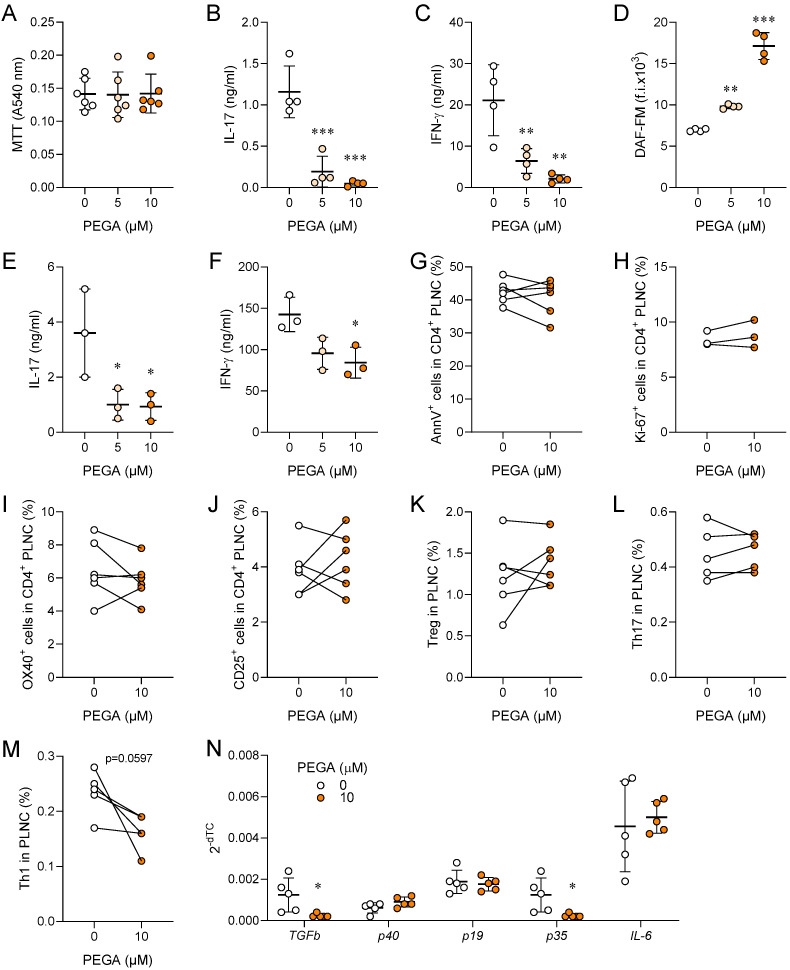
Immunomodulatory effects of PEGA on PLNC. Rats were immunized with myelin oligodendrocyte glycoprotein (MOG) 35-55+CFA (**A**–**F**) or with spinal cord homogenate (SCH) (**G**–**N**). PLNC were isolated on day 6 p.i. and stimulated with MOG35-55 (**A**–**D**), or with MBP (**G**–**N**), or with phorbol 12-myristate 13-acetate (PMA) and ionomycin in the presence of brefeldin A for 4 h before staining (**L**,**M**). CD4+ cells were purified from PLNC obtained on day 6 p.i. and stimulated with anti-CD3 and anti-CD28 antibodies (**E**,**F**). The cells were cultivated in the absence (0) or presence of PEGA for 24 h and cell viability was determined with 3-(4,5-dimethylthiazol-2-yl)-2,5-diphenyltetrazolium bromide (MTT) (**A**), Cytokine levels were determined in cell culture supernatants by ELISA (**B**,**C**,**E**,**F**), 4-amino-5-methylamino-2′,7′-difluorofluorescein diacetate (DAF-FM) was measured by fluorimetry (**D**), Apoptosis was determined by Annexin V staining (**G**), proliferation was measured by Ki67 staining (**H**), activation was assessed by CD25 (**I**) and OX40 staining (**J**) in CD4^+^ T cells by flow cytometry. Treg were identified as CD4^+^CD25^+^Foxp3^+^ cells (**K**), Th17 as CD4^+^IL-17^+^ cells (**M**), and Th1 as CD4^+^IFN-γ^+^ cells (**M**) by flow cytometry. Relative levels of cytokines mRNA were determined by “real-time” RT-PCR (**N**). Individual values of 3 (**E**,**F**,**H**), 4 (**B**–**D**), 5 (**L**–**N**), or 6 (**A**,**G**,**I**–**K**) samples are presented. In addition, the mean values +/− SD are given (**A**–**F**) One-way ANOVA followed by Tukey’s multiple comparison test (**A**–**F**), paired Student’s *t*-test (**G**–**M**), and unpaired Student’s *t*-test (**N**), * *p* < 0.05, ** *p* < 0.01, *** *p* < 0.001, vs. 0.

PLNC were obtained from rats 6 d.p.i., i.e., in the inductive phase of EAE. PEGA (5 μM and 10 μM) did not affect PLNC viability (Figure 1A), but it potently inhibited myelin basic protein (MBP)-induced release of IL-17 and IFN-γ from the cells (Figure 1B,C). At the same time, NO production was increased in PLNC (Figure 1D). The inhibitory effect of PEGA on IL-17 and IFN-γ was also shown in CD4^+^ cells purified from PLNC (Figure 1E,F). PEGA did not affect apoptosis and proliferation of CD4^+^ cells (Figure 1G,H), or the proportion of OX40^+^ or CD25^+^ CD4^+^ cells, i.e., activated CD4^+^ T cells (Figure 1I,J) among PLNC. Additionally, PEGA did not modulate the proportion of Treg and Th17 cells, but it decreased the proportion of Th1 cells among PLNC (Figure 1K–M). Furthermore, PEGA potently reduced mRNA expression of Th1-promoting IL-12 (p35/p40), and Th17-promoting TGF-β, but not Th17-promoting cytokines IL-6 and IL-23 (p19/p40) (Figure 1N).

### 2.2. PEGA Affects Spinal Cord Immune Cells (SCIC)

**Figure 2:**
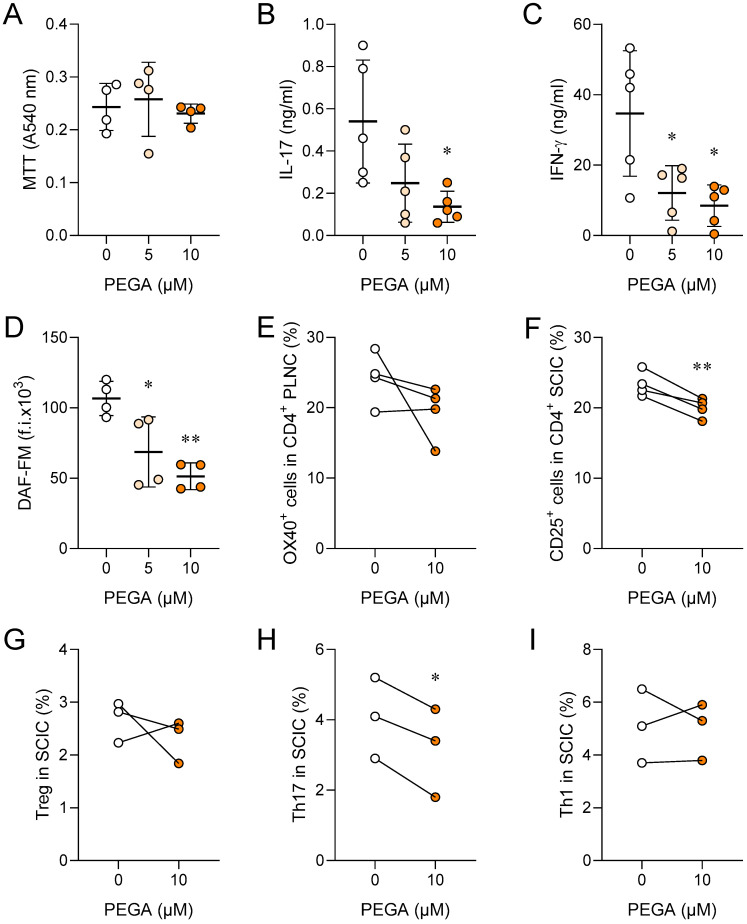
Immunomodulatory effects of PEGA on SCIC. Rats were immunized with SCH. SCIC were isolated on day 11 p.i. and cultivated in the absence (0) or presence of PEGA for 24 h, and cell viability was determined by MTT (**A**), cytokine levels were determined in cell culture supernatants by ELISA (**B**,**C**), DAF-FM was measured by fluorimetry (**D**), activation was assessed by OX40 (**E**) and CD25 staining (**F**) in CD4^+^ T cells by flow cytometry. Treg were identified as CD4^+^CD25^+^Foxp3^+^ cells (**G**). To determine Th17 (CD4^+^IL-17^+^) and Th1 (CD4^+^IFN-γ^+^) cells by flow cytometry, SCIC were stimulated with PMA and ionomycin in the presence of brefeldin A for 4 h before staining (**H**,**I**). Individual values of 5 (**B**,**C**), 4 (**A**,**D**–**F**) or 3 (**H**,**I**) samples are presented. In addition, the mean values +/− SD are given (**A**–**D**). One-way ANOVA followed by Tukey’s multiple comparison test (**A**–**D**), paired Student’s *t*-test (**E**–**I**), * *p* < 0.05, ** *p* < 0.01, *** *p* < 0.001, vs. 0.

SCIC were isolated from rats at the peak of EAE. PEGA did not affect their viability (Figure 2A); still, but significantly reduced the release of IL-17, IFN-γ, and NO from the cells (Figure 2B–D). PEGA also decreased the proportion of OX40^+^ or CD25^+^ CD4^+^ cells, i.e., activated CD4^+^ T cells (Figure 2E,F) among SCIC. Further, PEGA did not modulate the proportion of Treg and Th1 cells, but reduced the proportion of Th17 cells among SCIC (Figure 2G–I).

### 2.3. PEGA Affects Macrophages/Microglia

**Figure 3:**
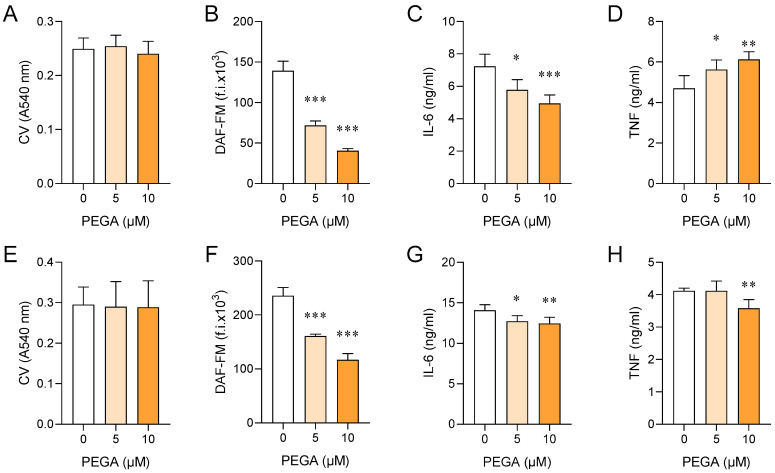
Effects of PEGA on macrophages and microglial cells. Lipopolysaccharide (LPS)-stimulated PM (**A**–**D**) or IFN-γ^+^LPS-stimulated BV2 cells (**E**–**H**) were untreated (0) or treated for 20 h with PEGA. Viability was determined by the crystal violet (CV) test (**A**,**E**), NO production was assessed by DAF-FM staining (**B**,**F**), IL-6 (**C**,**G**) and TNF (**D**,**H**) levels were determined in cell culture supernatants by ELISA. Data are presented as mean + SD obtained in 4 (**A**–**D**), 8 (**E**), 6 (**F**), or 5 (**G**,**H**) independent experiments. One-way ANOVA followed by Tukey’s multiple comparison test, * *p* < 0.05, ** *p* < 0.01, *** *p* < 0.001, 0 vs. PEGA.

Peritoneal macrophages (PM) were isolated from naïve rats. PEGA did not affect cell viability (Figure 3A), while it efficiently inhibited NO and IL-6, but upregulated TNF release from PM (Figure 3B–D). PEGA also did not affect BV2 cell viability, but inhibited NO, IL-6, and TNF release from these cells (Figure 3E–H).

### 2.4. PEGA Ameliorates EAE

**Figure 4:**
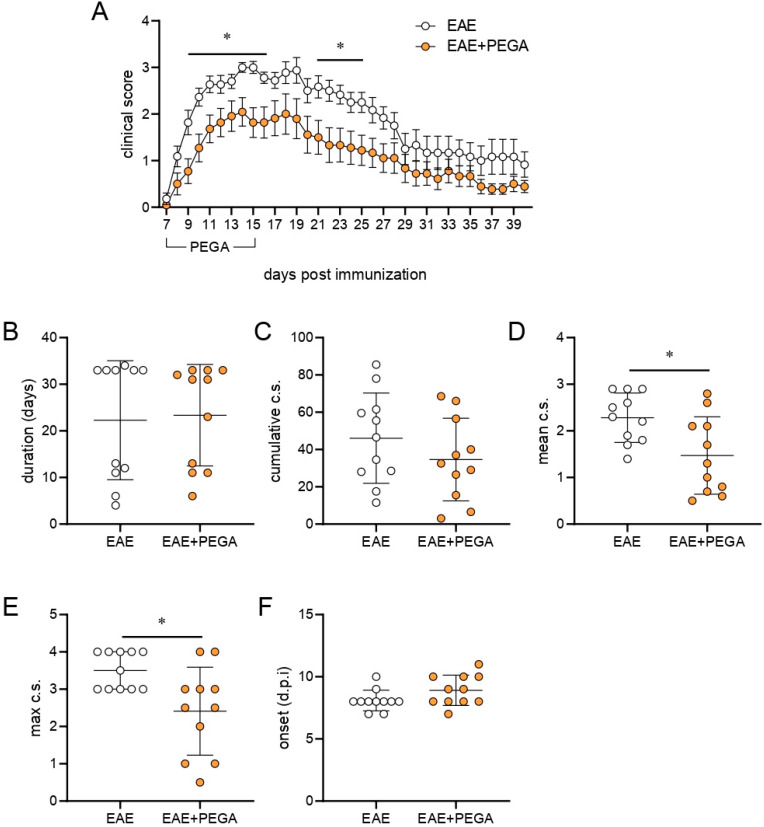

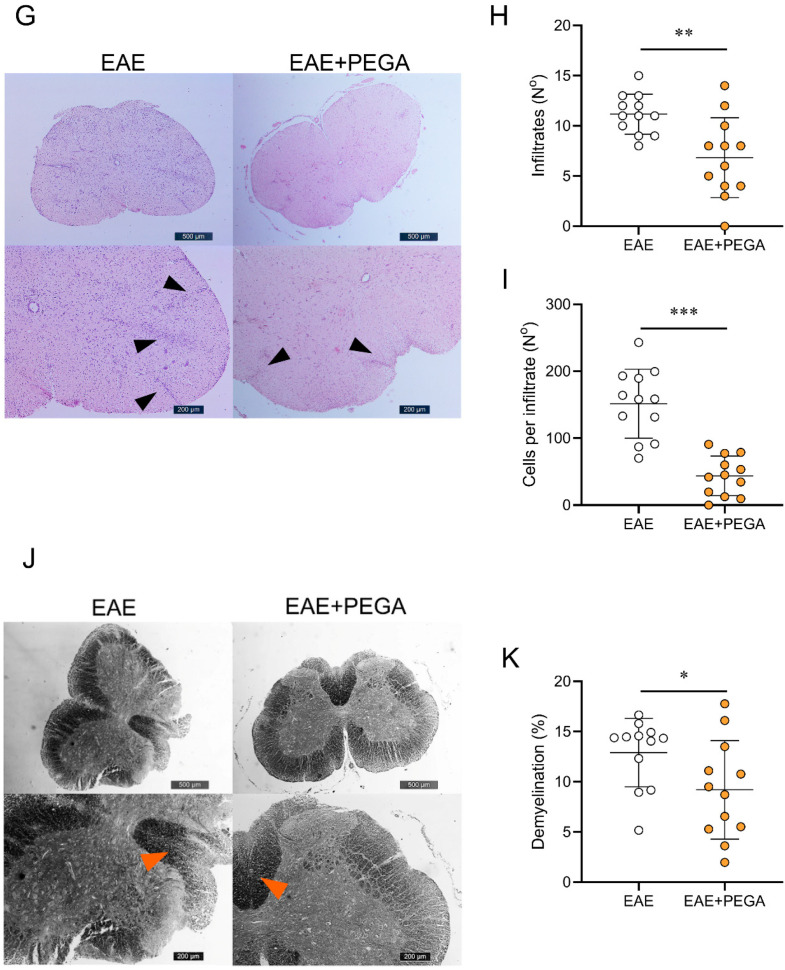
Effects of PEGA on EAE. PEGA or vehicle were administereddaily for 9 days, starting on day 7 p.i. Clinical signs were monitored daily (**A**) in vehicle-treated (EAE) and PEGA-treated (EAE+PEGA) rats. The duration of EAE (**B**), cumulative c.s. (**C**), mean c.s. (**D**), maximal c.s. (**E**), and day of onset (**F**) were calculated (n = 11 rats per group). Spinal cord sections from PEGA-treated or untreated rats were obtained at the peak of clinical signs in the EAE group and stained with H&E (**G**, representative micrographs, scale bar–500 µm, infiltrates are indicated by the arrows). The number of infiltrates per spinal cord section (**H**), and the number of cells per infiltrate (**I**) were determined on H&E-stained sections (three rats per group, four sections per rat). Demyelinated areas were visualized by Sudan black staining (**J**, representative micrographs, scale bar—500 µm) and quantified by ICY (three rats per group, four sections per rat) (**K**). Data are presented as mean +/− SD. Student’s *t*-test, * *p* < 0.05, ** *p* < 0.01, *** *p* < 0.001, EAE vs. EAE + PEGA.

The effect of PEGA on CNS autoimmunity was explored in rat EAE. PEGA, which was administered daily for 9 consecutive days starting on day 7 (one day before the expected first clinical manifestations), ameliorated EAE in rats (Figure 4A). It significantly reduced the clinical parameters of EAE, mean clinical score (c.s.), and maximal c.s. (Figure 4D,E), while it had a limited effect on the cumulative c.s., (Figure 4C), and no effect on disease duration and day of onset (Figure 4B,F). Additionally, the number of rats euthanized due to ethical reasons was lower in the EAE+PEGA group in comparison to the EAE group, with two and five rats, respectively. These effects were paralleled in reduction of cell infiltration into the spinal cord by PEGA at the time of EAE peak, as determined by hematoxylin and eosin (H&E) staining (Figure 4G–I). Further, PEGA reduced demyelination in spinal cords, as determined by Sudan black staining (Figure 4J,K).

## 3. Discussion

In this work, we used a rapid microwave-assisted process to develop PEGA, toward its evaluation against EAE. We found that PEGA reduces the inflammatory potency of T cells and macrophages/microglia, by downregulating their ability to produce/release IL-17 and IFN-γ, and IL-6 and NO, respectively. Importantly, it effectively inhibits ongoing autoimmune responses against the CNS and ameliorates EAE.

The observed effect of PEGA on the ability of T cells in the lymph nodes and spinal cord to generate IFN-γ and IL-17 is highly relevant for its beneficial influence on EAE. Injected CNS antigens are taken up by dendritic cells at the periphery and delivered to draining lymph nodes where they are presented to T cells. As a result, encephalitogenic T cells, mainly IFN-γ-producing Th1 cells and IL-17-producing Th17 cells, are activated [14,15]. These cells invade the CNS, where they are reactivated by local antigen-presenting cells [16] and consequent full-blown inflammation is established. IFN-γ and IL-17 have a major role in the inflammatory response and consequent destruction of CNS tissue [17]. 

Furthermore, the limitation of NO release by immune cells in the CNS is also important for the beneficial effects of PEGA in EAE. Namely, much of the CNS tissue destruction during neuroinflammation comes as a consequence of the deleterious activities of NO and its derivative peroxynitrite [18,19,20]. Our previous studies have clearly demonstrated that overproduction of NO within the CNS is detrimental in EAE in DA rats [21,22]. Hence, the observed negative effects of PEGA on NO release from SCIC, macrophages, and microglia are highly applicable for understanding of the PEGA-imposed amelioration of EAE in our model. On the other hand, NO, which is produced at the periphery, especially in lymph nodes, affects the activation of encephalitogenic T cells and therefore largely contributes to the resistance to EAE [23,24]. From this point of view, the results of our study showing the potentiation of NO release in PLNC by PEGA, imply that this activity of the compound may also contribute to the limitation of EAE. Moreover, the production of inflammatory cytokines TNF and IL-6 by macrophages/microglia has been implicated in the pathogenesis of CNS autoimmunity [25,26]. Therefore, the inhibitory influence of PEGA on the release of IL-6 in macrophages/microglia and TNF in microglia may contribute to the amelioration of EAE by the agent. The observed inhibitory effects of PEGA on NO, TNF, and IL-6 are in accordance with the previously reported influence of GA and GA-like compounds on immune cells in vivo and in vitro [27,28,29,30]. Interestingly, TNF release from macrophages was increased under the influence of PEGA. Although TNF is generally considered pro-inflammatory and supportive of T cell activation, this cytokine can also contribute to the regulation of immune responses, and specifically to the reduction of T cell activation [31]. Moreover, TNF has been shown to support Treg [32]. Thus, we could speculate that the increase of TNF at the periphery, where macrophages are among the major producers of this cytokine, is a limiting factor in encephalitogenic T cell activation. On the other hand, inhibition of TNF within the CNS, where microglia dominate over macrophages would reduce the extent of tissue damage. Still, it has to be emphasized that TNF production in macrophages was induced by LPS in our experiments, and that it has been reported that the regulation of TNF generation in macrophages is different under the influence of LPS vs. co-cultivation with T cells [33]. Thus, additional research, particularly in vivo, is needed to make the conclusion on the contribution of TNF modulation by PEGA to the observed beneficial effects of the agent in CNS autoimmunity.

Although positive effects of GA in EAE have already been reported [11], our study has some advantages. Namely, GA was applied to EAE mice during the inductive phase of the disease. Actually, the application started one day before the immunization, and lasted for 10 days. The advantage of our study was that the application of PEGA started one day before the clinical signs of EAE, that is, in the effector phase of EAE. Thus, this approach more closely resembles pharmaceutical intervention in multiple sclerosis. Nevertheless, it has to be stated that the ideal parallel to therapeutic intervention in multiple sclerosis would be the use of PEGA in rats with already clinically established EAE. Such a study would certainly further test the potential of PEGA as a therapeutic agent in CNS autoimmunity. Furthermore, GA dissolved in corn oil was administered intraperitoneally. This method of corn oil application induces inflammatory changes in the peritoneal cavity [34], and could affect the immune response in the mice. PEGA was applied subcutaneously, and corn oil effects were avoided in this way. On the other hand, this way of application led to an increase in the PEGA dose. Indeed, the PEGA dose was much higher, 20 mg/kg vs. 4 mg/kg of GA, yet no side effects were observed. Importantly, the in vitro effects of PEGA were achieved with 5 μM and 10 μM concentrations in our study, while 80 μM GA was applied in the study by Abdullah and colleagues. Thus, we can conclude that PEGA is more potent in its anti-inflammatory effects in comparison to its parent compound. Further, it has to be noted that PEGA was studied in DA rats immunized with SCH without complete Freund’s adjuvant (CFA). This system is advantageous compared to classical MOG_35-55_-induced EAE in C57BL/6 mice, because it excludes the use of CFA. This is very important having in mind various confounding effects that CFA has in EAE, particularly affecting the model’s capacity for translation of the obtained results to human studies [35]. However, to make the conclusion on the comparative effectiveness of PEGA and GA in CNS autoimmunity, a study of the direct comparison of the effects of the compounds in EAE is needed. Since GA acts as an AhR ligand in the EAE setting [11], it would be important to explore whether PEGA also activates AhR. Given the importance of AhR signaling to gut immune cells [36] and the importance of gut immune cells in the pathogenesis of EAE and multiple sclerosis [37,38,39], it would be of great importance to explore whether the effects of PEGA on CNS autoimmunity can also be achieved by oral administration, and what effect PEGA has on gut immune cells. To conclude, having in mind the efficiency of PEGA in EAE, as well as similarities in the pathogenesis of EAE and multiple sclerosis [15,17,40], further studies on the pharmaceutical potency of PEGA in the context of CNS autoimmunity are reasonable.

## 4. Materials and Methods

### 4.1. Synthesis and Application of PEGA

**Figure 5:**
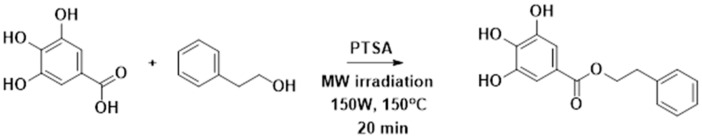
Synthetic procedure of PEGA.

**Figure 6:**
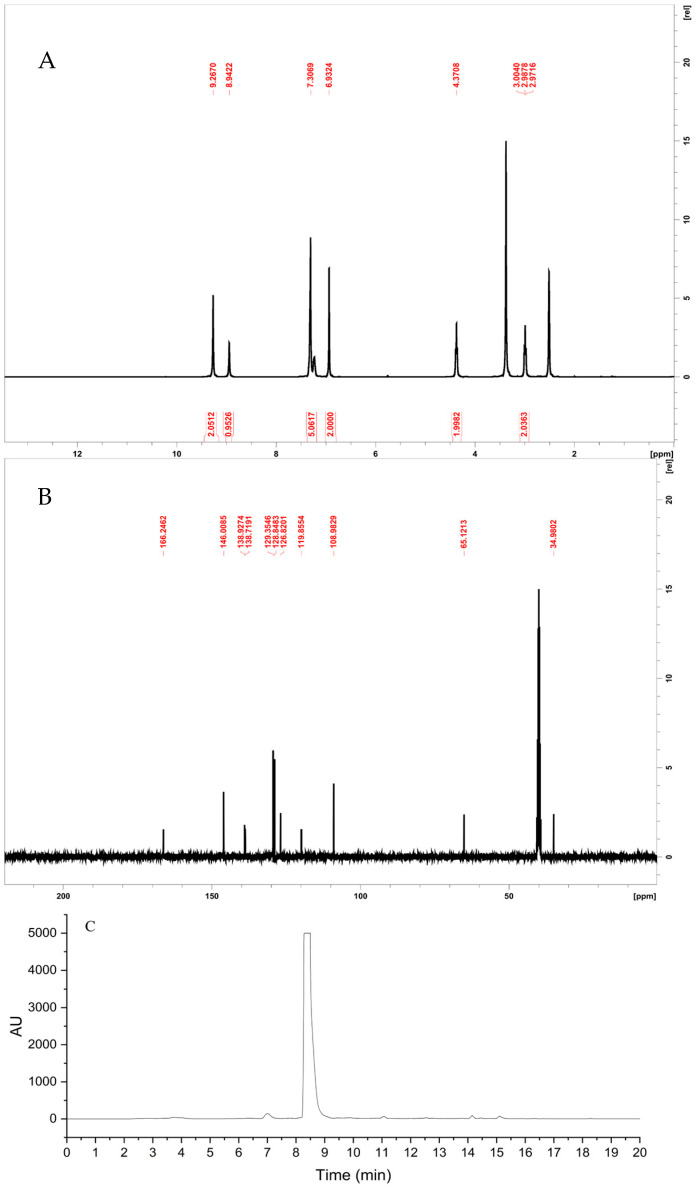
Characterization of PEGA. (**A**) ^1^H−NMR spectra of PEGA. (**B**) ^13^C−NMR spectra of PEGA. (**C**) HPLC chromatogram of PEGA.

The chemical synthesis of PEGA was performed through an esterification reaction between the carboxylic acid moiety of gallic acid and phenethyl alcohol, by establishing a microwave-assisted protocol (Figure 5). The vessel was capped and irradiated with 150 W at 150 °C for 20 min. The crude product was diluted with DCM and the solution was extracted with an aqueous solution of sodium hydroxide 1N. The organic and aqueous layers were separated. The aqueous layers were combined and then acidified with hydrochloric acid 3N at which point the clear solution turned cloudy white from brown. The acidified aqueous layer was extracted by DCM. The organic layers were washed with NaHCO_3_ (aq), brine, and then dried over sodium sulfate. The solvent was then removed to afford a brown crude sludge. The crude product was purified using HPLC, to afford the desired compound at a yield of 44%. %. ^1^H NMR (400 MHz, DMSO- _d6_), δ (ppm), 9.26 (br s, 2H), 8.94 (br s, 1H), 7.30 (m, 5H), 6.93 (s, 2H), 4.37 (t, J = 6.7 Hz, 2 H), 3.00–2.97 (t, J = 6.7 Hz, 2H). ^13^C NMR (400 MHz, DMSO _d6_), δ (ppm), 166.24, 146.00, 138.92, 138.71, 129.35, 128.84, 126.82, 119.85, 108.98, 65.12, 34.98. The desired product was characterized by ^1^H/^13^C NMR and the purity was determined to be 99.8% (Figure 6). For in vivo application, PEGA was dissolved in DMSO (Santa Cruz Biotechnology, Dallas, TX, USA) at 500 mg/mL, and then diluted to a working concentration in sesame oil (Linum, Čonoplja, Serbia). For in vitro application, PEGA was dissolved in DMSO at 50 mM, and then diluted to a working concentration in the cell culturing medium. 

### 4.2. Experimental Animals, EAE, and PEGA Application

Five to seven months old Dark Agouti (DA) rats of both sexes were used in the experiments. Rats were bred and maintained in the animal facility of the Institute for Biological Research “Siniša Stanković”. The housing of the rats was performed under controlled environmental conditions, with three to five rats in the same cage. Animal experiments were approved by the Veterinary Administration, Ministry of Agriculture, Forestry and Water Management, Republic of Serbia (No 323-07-12374/2021-05). Immunization was performed with DA rat spinal cord homogenate in phosphate-buffered saline (PBS, 50% *w*/*v*), as previously described [13]. A total of 100 μL of PEGA (20 mg/kg) or DMSO in sesame oil was applied subcutaneously into the upper back of EAE+PEGA or EAE rats, respectively. Age- and sex-matched rats were randomly distributed into EAE+PEGA and EAE groups. Daily treatment started on day 7 post-immunization (p.i.) and lasted for 9 days for monitoring the effects of PEGA on the clinical course of EAE. No side effects were observed in the treated rats. The rats were monitored daily for clinical signs of EAE, as described previously [13]. Rats reaching score 4 (moribund state) were euthanized due to ethical reasons. Cumulative clinical score was calculated as a sum of daily c.s. Mean c.s. was calculated as cumulative c.s. divided by the duration. For the isolation of cells from the spinal cord and for spinal cord histology, rats were sacrificed at the time of disease peak in the EAE group (11–14 days p.i., c.s. 2.5–3.5). For some experiments specified below, rats were immunized with 0.5 mg/mL MOG35-55 peptide (SB-Peptide, Saint Egrève, France) mixed with an equal volume of complete Freund’s adjuvant (CFA, Difco, Detroit, MI, USA). Rats were injected subcutaneously into the hind hock with 100 μL of MOG + CFA.

### 4.3. Histological Assessment of EAE

Hematoxylin and eosin staining was performed on spinal cord tissue sections to detect inflammatory infiltrates, as described previously [35]. Sudan black staining was performed to detect demyelinating regions in the spinal cord sections, as described previously [41]. Quantification of infiltrates, cells per infiltrate, and demyelinated areas were performed on twelve spinal cord tissue sections obtained from three rats per group (four sections per rat). ICY software (BioImage Analysis Lab, Institut Pasteur, Paris, France) was used for the quantification of demyelination.

### 4.4. Isolation of Cells and Cell Cultures 

Popliteal lymph node cells, i.e., cells of lymph nodes draining the site of immunization, were obtained from immunized rats on days 6 p.i. For RT-PCR and flow cytometry, PLNC were obtained from rats immunized with SCH, while for ELISA and NO determination, PLNC were obtained from MOG35-55-immunized rats. Spinal cord immune cells were isolated from SCH-immunized rats on days 11-14 p.i., as previously described [14]. CD4+ cells were purified from PLNC obtained from MOG35-55-immunized rats by magnetic separation with biotin-conjugated antibody specific for rat CD4 (BD Pharmingen, San Diego, CA, USA) and IMagSAv Particles Plus (BD Biosciences, San Jose, CA, USA). Peritoneal macrophages were obtained from naïve rats by peritoneal lavage, as described previously [42]. BV2 cells were the kind gift of Dr. Alba Minelli (Università degli Studi di Perugia, Perugia, Italy).

PLNC were grown in RPMI-1640 medium (Capricorn Scientific, Ebsdorfergrund, Germany) supplemented with 2% rat serum. CD4+ cells, SCIC, and PM were cultivated in RPMI-1640 medium supplemented with 5% fetal bovine serum (FBS, Sigma-Aldrich, St. Louis, MO, USA), while BV2 cells were cultivated in RPMI-1640 with 10% FCS. CD4+ cells were seeded in 96-well plates, while the other cells were grown in 24-well plates (Sarstedt, Nümbrecht, Germany). Exceptionally, for NO determination, the cells were seeded in 96-well optical bottom plates (Nunc, Rochild, Denmark). All cultivations were performed at 37 °C in a humidified atmosphere containing 5% CO_2_.

PLNC were stimulated with myelin basic protein (10 μg/mL, guinea pig MBP, a kind gift from Professor Alexander Flügel, University of Göttingen, Germany) for RT-PCR, or with MOG35-55 (10 μg/mL) for ELISA and NO determination. A total of 5 × 10^6^/mL/well were seeded for RT-PCR and ELISA, while 2 × 10^5^/200 μL/well were seeded for NO measurement. For SCIC, 5 × 10^6^/mL/well for ELISA and 2 × 10^5^/200 μL/well for NO determination were cultivated without stimulation. CD4+ cells (1 × 10^6^/200 μL/well) were grown in plates pre-coated with anti-CD3 and anti-CD28 antibodies (both at 1 μg/mL, BD Biosciences). PM (obtained from 2 × 10^6^ peritoneal cells/well for ELISA, or from 2 x 10^5^/well for NO determination) were stimulated with 10 ng/mL LPS (Sigma-Aldrich). For BV2, 5 × 10^5^/mL/well for ELISA or 2 × 10^5^/200 μL/well for NO measurement were stimulated with 10 ng/mL of recombinant mouse IFN-γ (Peprotech, Rocky Hill, NJ, USA) and 10 ng/mL of LPS. All cultivations were performed in the absence or presence of PEGA and lasted for 24 h, except for NO determination where cultivation lasted for 20 h. Subsequently, cell culture supernatants were collected and kept frozen until assayed in ELISA, while cells were subjected to cell viability assays, RT-PCR, NO measurement, or flow cytometry. For intracellular cytokine detection, PLNC (without MBP) and SCIC were treated with a cell stimulation cocktail containing PMA, ionomycin, and protein transport inhibitor (eBioscience, San Diego, CA, USA) for the last 4 h of cultivation.

### 4.5. Cell Viability Assays

The viability of the PLNC and SCIC was assessed by the MTT assay, while PM and BV2 viability was assessed by the CV test, as described previously [15 Miljkovic et al., 2015]. The absorbance was measured at 540 nm with a correction at 670 nm on an automated microplate reader (Synergy H1, Agilent BioTek, Santa Clara, CA, USA).

### 4.6. ELISA

Cytokine concentration in cell culture supernatants was determined by sandwich ELISA using MaxiSorp plates (Nunc). For cytokine detection, anti-cytokine paired antibodies were used according to the manufacturer’s instructions (rat IFN-γ rat/mouse IL-17, mouse IL-6, rat TNF—Thermo Fisher Scientific, Waltham, MA, USA; rat IL-6—R&D Systems, Minneapolis, MN; mouse TNF—Abcam, Cambridge, UK). The antibodies were as follows: anti-rat IFN-γ purified mouse monoclonal (DB1), anti-rat IFN-γ biotinylated rabbit polyclonal, anti-mouse/rat IL-17A purified rat monoclonal (eBio17CK15A5), anti-mouse/rat IL-17A biotinylated rat monoclonal (eBio17B7), anti-mouse IL-6 purified mouse monoclonal (MP5-20F3), anti-mouse IL-6 biotinylated mouse monoclonal (MP5-32C11), anti-rat IL-6 purified mouse monoclonal (#53325), anti-rat IL-6 biotinylated goat polyclonal IgG, anti-mouse TNF purified rabbit monoclonal (EPR16803-2), anti-mouse TNF biotinylated rabbit monoclonal (EPR16803-84), anti-rat TNF purified Armenian hamster monoclonal (TN3-19.12), and anti-rat TNF biotinylated rabbit polyclonal IgG. The absorbance was recorded at 450 nM with a correction at 670 nm using a multiplate reader Synergy H1. Samples were analyzed in duplicates and the results were calculated using standard curves based on known concentrations of the recombinant rat IFN-γ and rat IL-17 (Peprotech), mouse IL-6 and rat TNF (Thermo Fisher), rat IL-6 (R&D Systems), and mouse TNF (Abcam). For all ELISA tests, the lower limit of detection was 30 pg/mL, while the upper limit of detection was 10 ng/mL.

### 4.7. NO Detection

DAF-FM staining was used for the determination of NO production. The cells were treated with DAF-FM acetate (Thermo Fisher Scientific) in the absence or presence of PEGA for 20 h (DAF-FM). Fluorescence intensity (f.i.) was detected with a multiplate reader Synergy H1, using excitation at 510 nm and emission at 540 nm for DAF-FM.

### 4.8. Cytofluorimetry

PLNC and/or SCIC were stained with the following antibodies: FITC- or PE-conjugated anti-CD4 (mouse monoclonal OX35, eBioscience), PE-conjugated anti-CD25 (mouse monoclonal OX39, eBioscience), APC-conjugated anti-CD134 (mouse monoclonal OX40, BD Pharmingen), PerCP-Cy5.5-conjugated anti-IL-17 (rat monoclonal eBio17B7, BDPharmingen), FITC-conjugated anti-IFN-γ (rabbit polyclonal, eBioscience), PerCP-Cy5.5-conjugated anti-Foxp3 (rat monoclonal FJK-16s, eBioscience), and FITC-conjugated anti-Ki-67 (rat monoclonal SolA15, eBioscience). Intracellular staining for cytokines, FoxP3 and Ki67, was performed according to the procedure suggested by the manufacturer (eBioscience), using Foxp3/transcription factor fixation/permeabilization concentrate and diluent, intracellular fixation & permeabilization buffer set, and permeabilization buffer (all from eBioscience), as appropriate. Appropriate isotype control antibodies were used as needed to set gates for cell marker positivity. Typically, the proportion of isotype control antibody-stained cells was < 1%. FITC-conjugated annexin V was used for the detection of apoptotic cells in accordance with the manufacturer’s instructions (eBioscience). The acquisition of the samples was performed on a BD FACS Aria III cell sorter (BD Biosciences) and analyzed by FlowJo software v.10 (BD Biosciences). Results of cytofluorimetry are presented as the proportion of cells bound by an appropriate antibody.

### 4.9. “Real-Time” RT-PCR

Total RNA was isolated from cells using a mi-Total RNA Isolation Kit (Metabion, Martinsried, Germany) and reverse transcribed using random hexamer primers and MMLV (Moloney murine leukemia virus) reverse transcriptase according to the manufacturer’s instructions (Fermentas, Vilnius, Lithuania). Prepared cDNAs were amplified by using Maxima SYBR Green/ROX qPCR Master Mix (Fermentas, Vilnius, Lithuania) according to the recommendations of the manufacturer in a QuantStudio 3 real-time PCR system (Applied Biosystems, Foster City, CA, USA). Thermocycler conditions comprised an initial step at 50 °C for 5 min, followed by a step at 95 °C for 10 min, and a subsequent two-step PCR program at 95 °C for 15 s and 60 °C for 60 s for 40 cycles. The PCR primers (Metabion, Martinsried, Germany) were as follows: IL-6: 5′-GCC CTT CAG GAA CAG CTA TGA-3′; 5′-TGT CAA CAA CAT CAG TCC CAA GA-3′; p19: 5′-GGG AGA CTC AAC AGA TGC CT-3′; 5′-GCA CTA AGG GCT CAG TCA GA-3′; p35: 5′-CAT CAC ACG GGA CAA AAC CA-3′; 5′-AGG CAC AGG GTC ATC ATC AA-3′; p40: 5′-CAC ATC TGC TGC TCC ACA AG-3′; 5′-CAA GTC CGT GTT TCT GTG CA-3′; transforming growth factor-beta (TGF-β): 5′-CCC TGC CCC TAC ATT TGG A-3′; 5′-ACG GTG ATG CGG AAG CAC-3′; GAPDH: 5′- ACA TCA TCC CTG CAT CCA CT-3′; 5′-GGG AGT TGC TGT TGA AGT CA-3′. Accumulation of PCR products was detected in real-time, and the results were analyzed with QuantStudio 3 software. Relative RNA expression is presented as 2−dCt, where dCt is the difference between Ct values of a gene of interest and the endogenous control (β-actin).

### 4.10. Statistical Analysis 

Sample size for each of the analyses was selected based on our previous studies, using the Biomath^®^ power calculator (http://biomath.info/power). Statistical analysis was performed using GraphPad Prism 8 software (GraphPad Software, San Diego, CA, USA). The significance of the differences between the groups was determined using a two-tailed Student’s *t*-test or one-way ANOVA followed by Tukey’s post hoc test as indicated in the figure legends. A *p*-value less than 0.05 was considered statistically significant.

## Data Availability

The data presented in this study are available on request from the corresponding author.
